# DNA methylation clocks as a predictor for ageing and age estimation in naked mole-rats, *Heterocephalus glaber*

**DOI:** 10.18632/aging.102892

**Published:** 2020-03-03

**Authors:** Robert Lowe, Amy F. Danson, Vardhman K. Rakyan, Selin Yildizoglu, Frédéric Saldmann, Melanie Viltard, Gérard Friedlander, Chris G. Faulkes

**Affiliations:** 1 The Blizard Institute, Barts and The London School of Medicine and Dentistry, Queen Mary University of London, London, UK; 2 Centre for Genomic Health, Queen Mary University of London, London, UK; 3 Fondation pour la Recherche en Physiologie, Brussels, Belgium; 4 Service de Physiologie et Explorations Fonctionnelles, Hôpital Européen Georges Pompidou, Assistance Publique-Hôpitaux de Paris, Paris, France; 5 Université Paris Descartes, Faculté de Médecine, Paris, France; 6 INSERM UMR_S1151 CNRS UMR8253 Institut Necker-Enfants Malades (INEM), Paris, France; 7 School of Biological and Chemical Sciences, Queen Mary University of London, London, UK

**Keywords:** naked mole-rat, *Heterocephalus glaber*, methylation clocks, epigenetic clocks, ageing

## Abstract

The naked mole-rat, *Heterocephalus glaber* (NMR), the longest-lived rodent, is of significance and interest in the study of biomarkers for ageing. Recent breakthroughs in this field have revealed ‘epigenetic clocks’ that are based on the temporal accumulation of DNA methylation at specific genomic sites. Here, we validate the hypothesis of an epigenetic clock in NMRs based on changes in methylation of targeted CpG sites. We initially analysed 51 CpGs in NMR livers spanning an age range of 39-1,144 weeks and found 23 to be significantly associated with age (p<0.05). We then built a predictor of age using these sites. To test the accuracy of this model, we analysed an additional set of liver samples, and were successfully able to predict their age with a root mean squared error of 166 weeks. We also profiled skin samples with the same age range, finding a striking correlation between their predicted age versus their actual age (R=0.93), but which was lower when compared to the liver, suggesting that skin ages slower than the liver in NMRs. Our model will enable the prediction of age in wild-caught and captive NMRs of unknown age, and will be invaluable for further mechanistic studies of mammalian ageing.

## INTRODUCTION

The naked mole-rat (*Heterocephalus glaber*) is arguably unique among mammals in the extent of its social insect-like behaviour, first highlighted by Jarvis [1]. In more recent years it has also gained prominence as an important non-model organism for the study of longevity [2–5], and other extraordinary aspects of its biology that result from adaptations to the challenges of an extreme subterranean niche [6, 7]. Although little more than mouse-sized (mean body mass is around 35g), naked mole-rats are the longest-lived rodent with a maximum possible lifespan exceeding 31 years [5]. Furthermore, they resist all of the normal signs of ageing and are thus emerging as an important non-model organism for the study of longevity and healthspan. These unusual mammals uniquely do not show an increase in age-specific hazard of mortality, in defiance of Gompertz’s law [8]. They also show no decreased physiological capacity with age, maintaining vascular elasticity, cardiac function, gastrointestinal function, glucose tolerance, and reproductive capacity well into the third decade of life [2, 9, 10], and resist sarcopenia, the progressive loss of skeletal muscle with age [11]. Because of these traits, the naked mole-rat is of particular significance and interest in the study of biomarkers for ageing. Recently, breakthroughs in this field have indicated the presence of ‘epigenetic clocks’, mainly in studies of human and mouse tissues [12–15], but also in canids [16, 17], and humpback whales [18]. These are based on the temporal accumulation of DNA methylations at specific ageing-associated differentially methylated positions (aDMPs). Such CpG sites at which DNA methylation dynamics show significant correlations with age can potentially enable accurate age estimates for tissues across the life span of an individual, and it has been shown in humans that most tissues and organs from the same body exhibit broadly similar epigenetic ages [19].

In an analysis of aDMPs in six different mammals, including long and short-lived dog breeds, Lowe et al*.* [17] found a strong negative relationship between rate of change of methylation levels at aDMPs and lifespan. This study also identified 30 aDMPs in the naked mole-rat liver, that clustered in 12 different targeted aDMP regions, providing a potential molecular readout for aging in this species. A challenge for research on long-lived non-model organisms such as the naked mole-rat, especially when studying wild caught animals, is the determination of age (in the absence of birth and life history data). In some cases, this problem also applies to captive populations where pedigree data is not available. Crude categorical estimates are sometimes possible based on tooth wear, for example in other African mole-rats (*Cryptomys* and *Fukomys*; [20]), but these only differentiate relative age classes, rather than attempting to assign an absolute age estimate. Here, we aim to (i) consolidate and validate these initial results for the naked mole-rat, examining further samples across a wide age range in both liver and skin, and (ii) develop a method that will enable naked mole-rat aDMPs to be used to estimate age in animals of unknown provenance.

## RESULTS

**Figure 1:**
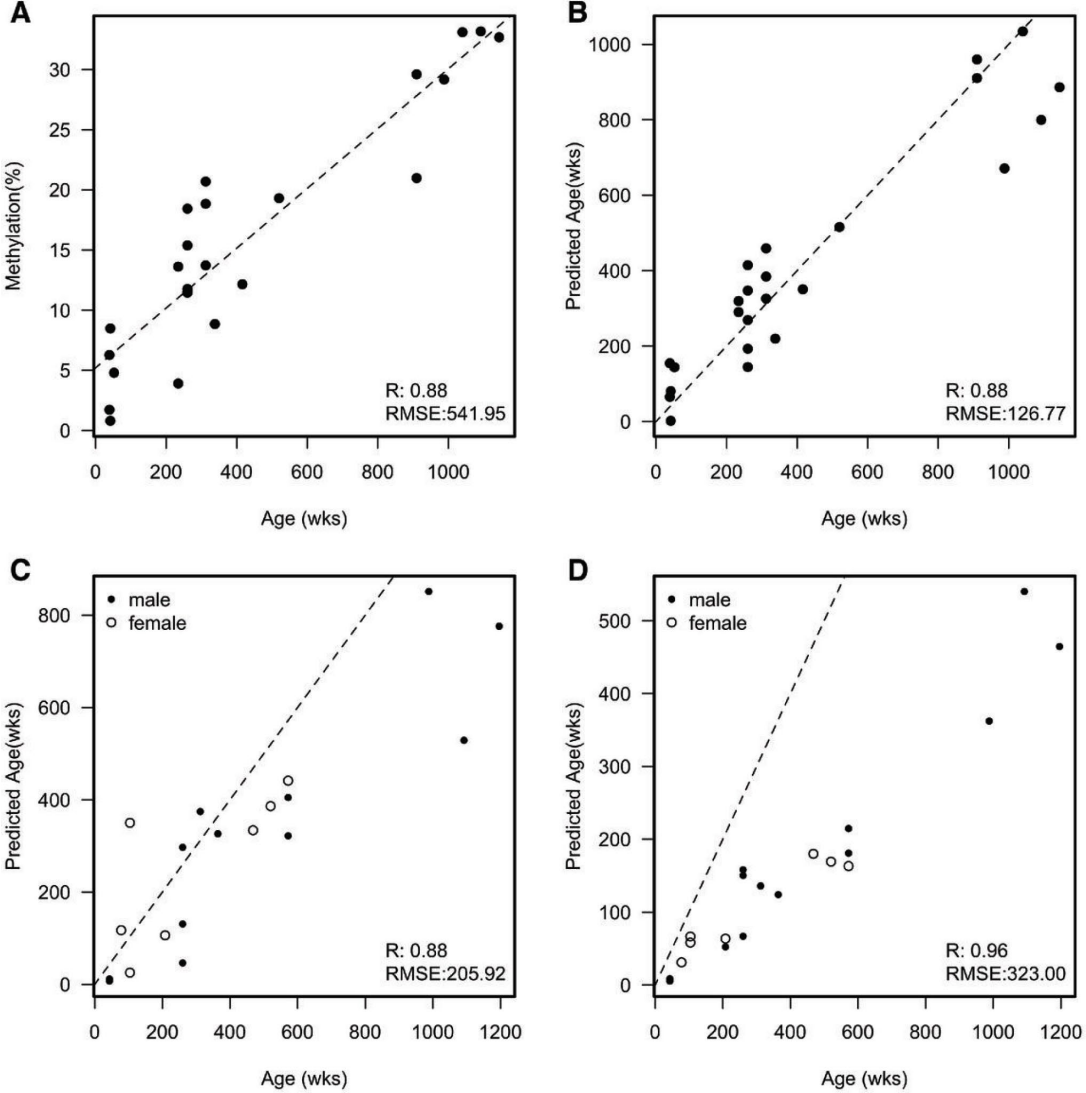
(**A**) Example of a single CpG that correlates with the age of each naked mole-rat. Dashed line is a fitted linear model; RMSE = Root Mean Square Error. Mean absolute deviation (MAD) = 412.282 and median absolute error (MAE) = 400.198; (**B**) A scatterplot of the predicted age of each naked mole-rat liver sample against the actual age in weeks from an initial sample set. The predicted age was calculated by removing the sample and fitting to the remaining samples. Dashed line represents y=x (e.g. perfect prediction). MAD = 96.882 and MAE = 120.840; (**C**) A scatter plot of the predicted age of a second set of naked mole-rat livers against the actual age in weeks. Dashed line represents y=x (e.g. perfect prediction). MAD = 118.941 and MAE = 126.883; (**D**) A scatter plot of the predicted age of skin samples against the actual age in weeks. Dashed line represents y=x (e.g. perfect prediction). MAD = 219.113 and MAE = 252.874.

To create a method for predicting the age of naked mole-rats based on changes in methylation, we initially sampled 24 naked mole-rat livers spanning an age range from 39 weeks to 1,144 weeks (approximately ten months to 22 years; Supplementary Table 1). We performed a targeted sequence-based method to determine the methylation of individual CpGs across the genome. These targeted regions were selected by mapping existing regions within the human genome, known to be associated with age, to the naked mole-rat genome (https://www.ncbi.nlm.nih.gov/pubmed/25172923). In total we selected 12 regions/primer pairs spanning a total of 51 different CpGs (Supplementary Table 2). Of these 51 different sites 23 (45%) were found to be associated with age (p-value < 0.05; Supplementary Table 3). The top hit (JH602136:8746439) showed a strong correlation (R=0.88) with age (Figure 1A) with a root mean square error of 541.95 weeks.

Utilising a similar methodology to Horvath [19] in producing his multi-tissue age prediction in humans, we built a predictor of age using the 23 sites that showed an association with age in the naked mole-rat. This uses elastic net regression which incorporates all 23 CpGs in a multivariate analysis. To validate this model, we performed a leave one out cross validation in which we remove a single sample to fit the parameters of our model and then predict the age of this single sample. We then repeated this until we had removed each sample and predicted its age. Utilising multiple CpGs in the model showed a slight improvement in correlation (0.89) but a large decrease in the RMSE (134.21) (Figure 1B). To further test whether this model could be useful, we sampled a further 19 livers with an age range 43 to 1,196 weeks (approximately ten months to 23 years; Supplementary Table 1). Using the model built from the initial 24 samples, we predicted the age of these new samples. As expected, given this was a different batch of samples both the R and RMSE dropped albeit only by a small amount (Figure 1C). Given the multi-tissue nature of some methylation changes within humans, we decided to test our model using a different set of tissue samples. We profiled a further 20 skin samples with the same age range of that of the 19 liver samples (19 of the 20 skin samples were from the same animals as the livers). Interestingly we found a striking correlation between the predicted age of these samples versus their actual age (R=0.93), however, this correlation was not along the identity line but showed a much lower predicted age than actual age suggesting that skin tissue ages slower than the liver. This change in rate accounts for the larger RMSE. To make this approach useable for other researchers we have provided the primer sequences that span the CpGs, and an online tool that can be downloaded as an NMR age predictor based on these aDMPs (https://github.com/ralowe/NMRAgePrediction; Supplementary Material).

## DISCUSSION

In an extensive study of naked mole-rats, we identify 23 CpGs in liver tissue that are significantly associated with age, and build a predictor of age model with a root mean squared error of 166 weeks, or approximately 10% of the published maximum possible lifespan (more than 31 years/1612 weeks; [5]). In profiling skin samples from the same individuals, we also found a striking correlation between the predicted age of these samples versus their actual age. Interestingly, when compared to the liver samples, this correlation showed a lower predicted age than actual age, suggesting that skin tissue ages more slowly than the liver in naked mole-rats. In humans, Horvath [19], found that most tissues from the same body exhibit broadly similar epigenetic ages. Other studies have showed that, while some age-associated changes in methylation are shared across tissues, others may be tissue-specific in humans [21, 22], mice (lung, liver, spleen and colon; [23]) and rats (liver and visceral adipose tissues; [24]). The relative proportions of age-associated epigenetic changes in the form of DNA methylations that are tissue-specific, compared to the amount that is general and non-specific remains a matter of debate. Zhu et al. [25] estimate that more than 70% is due to shared epigenetic drift across tissues, with the remainder down to tissue-specific and functionally important changes. The differences in ageing between liver and skin seen in the naked mole-rat may perhaps reflect the increased metabolic activity of the liver when compared to skin, and the fact that naked mole-rats are not exposed to UV radiation in captive or wild colonies. A variety of naked mole-rat tissues have been shown to resist the normal signs of senescence (see Lewis and Buffenstein [5] for review), including the liver where proteasome function increased or was maintained with age [26], and there was no accumulation of oxidative damage with age [27]. Although the ageing skin has not been investigated fully in naked mole-rats, their fibroblasts in culture are resistant to an array of toxins and stressors (compared with mice), including heavy metals, heat, and chemotherapeutic/DNA-damaging compounds [28, 29, 5]. Further, MacRae et al*.* [30] report that the naked mole-rat liver has higher expression of DNA repair genes, with significant upregulation of several DNA repair signaling pathways compared with the mouse. These observations of increased DNA repair in the naked mole-rat perhaps support the proposal of Field et al*.* [31], that long term maintenance of a steady state in dynamic chromatin (“chromostasis”) may slow the ticking of the epigenetic clock in long-lived species.

Horvath [19] suggests that understanding how and why the estimated epigenetic age differs across a group of individuals of the same chronological age could help to determine the impact of endogenous or exogenous stress factors on biological ageing. Humans suffering from Werner’s Syndrome, a condition that produces clinical signs of accelerated ageing also had associated epigenetic age acceleration and thus an increased DNA methylation age [32]. Other studies have shown that in humans, lifestyle factors including diet and physical activity, can have a positive association with epigenetic age acceleration i.e. a healthy lifestyle associates with a reduced epigenetic age [33]. Naked mole-rats are apparently unique among mammals in that they defy Gompertz’s Law in not showing increased risk of mortality with age [8]. Furthermore, there are no apparent sex or reproductive status differences in their maximum possible lifespan [3]. It is interesting that some variance in the percentage methylation is evident for liver tissue in Figure 1A, perhaps suggesting some differences in biological versus chronological age among these mole-rat samples.

The potential for a forensic use for epigenetic clocks to determine the unknown age of a sample or individual has also been noted for humans by Wagner [34]. Our model and online tool will enable the prediction of age in wild caught naked mole-rats and captive animals of unknown age. Given the evidence for associations between epigenetic age acceleration and factors that may influence longevity in humans, and that most clocks have focussed on application in humans [14] our study will be invaluable for further mechanistic and functional studies of mammalian ageing in non-model organisms such as the naked mole-rat.

## MATERIALS AND METHODS

Naked mole-rats were maintained in the Biological Services Unit at Queen Mary University of London, and tissues obtained from post-mortem specimens from animals free from disease in compliance with national (Home Office) and institutional procedures and guidelines. Because sample collection was from post-mortem material, additional local ethical approval was not required for this study. Samples of abdominal skin and liver were snap frozen in liquid nitrogen and transferred for storage at -80°C. Full details of animals and samples are provided in Supplementary Table 1.

### DNA extraction

DNA was extracted from tissues using the PureLink™ Genomic DNA kit (Thermo Fisher, Cat. K182002), as per the manufacturer’s instructions. Tissues were digested overnight at 55 °C using 180 μl PK buffer and 20 μl PK enzyme from the kit. DNA concentration was quantified using a High Sensitivity DNA Qubit® assay (Life Technologies, Cat. Q32851).

### Bisulfite PCR sequencing (Bis-PCR-Seq)

DNA from tissues was diluted to a concentration of 11 ng/μl and 45 μl of each sample was used for generation of targeted bisulfite sequencing data by the Genome Centre Facility at the Blizard Institute, Queen Mary University of London. DNA was bisulfite converted in a 96 well plate format using the EZ-96 DNA Methylation™ Kit (Zymo, Cat. D5003). Target amplification was performed using the FastStart High Fidelity PCR System, dNTPack (Sigma-Aldrich, Cat. 4738284001) in the 48.48 layout on the Fluidigm® C1 system (Fluidigm®, USA), a microfluidics platform. Library preparation was performed using the same kit including 4 μl of Access Array Barcode Library Primer and 1 μl of PCR product diluted 1:100. Libraries were sequenced with Illumina MiSeq sequencing using v2 chemistry (150 bp, paired-end). Primers used for targeted bisulfite sequencing are listed in Supplementary Table 2.

Raw FASTQ files were mapped to the reference hetGla2 using BISMARK (v0.16.3) [35] and Bowtie2 (v2.2.8) [36]. Reads that mapped outside of the targeted regions were discarded from analyses, and methylated and unmethylated counts for each CpG were calculated using the custom C++ program (https://bitbucket.org/ lowelabqmul/methylation-extractor/src/master/). Those CpGs with a coverage < 50× were also discarded from analyses. Data created for this manuscript are available from GEO with accession number GSE86059 (sample set 1) and GSE137957 (sample set 2), and provided in Supplementary File 1.

### Statistical analysis

The analysis model used elastic net regression incorporating all 23 CpGs in a multivariate analysis. Linear models and scatterplots were produced using R statistical software [37]. We have developed an online tool for analysis of similar NMR data (available at https://github.com/ralowe/NMRAgePrediction), with further information provided in the Supplementary Material).

## Supplementary Material

Supplementary Material

Supplementary Tables

Supplementary File 1
