# Supplementary material for: DNA methylation clocks as a predictor for ageing and age estimation in naked mole-rats, Heterocephalus glaber
Source: Aging (Albany NY). 2020 Mar 3;12(5):4394–406. doi: 10.18632/aging.102892 (PMC7093186; doi:10.18632/aging.102892)
Supplement: Supplementary Material [file aging-12-102892-s002..pdf]

SUPPLEMENTARY MATERIAL

NMRAgePrediction tool

A downloadable software package enabling users to predict age from NMR data obtained using the methodology reported in this paper is available at: (https://github.com/ralowe/NMRAgePrediction

Full instructions on use is included in the README file. The tool requires Python version 3.7, pip, numpy, and scipy to be installed on the local machine prior to running NMRAgePrediction. The tool uses a model based on our animals/samples of known age to predict age, based on the proportion of methylated sites at the NMR aDMPs we describe. The input data should be expressed as methylation beta values, i.e. the ratio of methylated and unmethylated alleles expressed between 0 and 1.0.
