# Supplementary material for: DNA methylation clocks as a predictor for ageing and age estimation in naked mole-rats, Heterocephalus glaber
Source: Aging (Albany NY). 2020 Mar 3;12(5):4394–406. doi: 10.18632/aging.102892 (PMC7093186; doi:10.18632/aging.102892)
Supplement: Supplementary Tables [file aging-12-102892-s003..pdf]

SUPPLEMENTARY TABLES

Supplementary Table 1. Sample details for the two sets of analyses, sample id corresponds to column headings in Supplementary File 1.

Sample (Set/id)	Anim/ No/	Colony	Age (yr)	Age (weeks)	Tissue
Set 1/a1	BM857	800	22	1144	Liver
Set 1/a2	M160	NN	20	1040	Liver
Set 1/a3	M224	Omega	17/5	910	Liver
Set 1/a4	M269	800	6	312	Liver
Set 1/a5	M576	800	5	260	Liver
Set 1/a6	M1	11A	0/8	42	Liver
Set 1/a7	M2	11A	0/8	42	Liver
Set 1/a8	M200	NN	19	988	Liver
Set 1/a9	M227	Omega	17/5	910	Liver
Set 1/a10	M065	800	5	260	Liver
Set 1/a11	M3	11A	1	52	Liver
Set 1/a12	M46	F(K)	0/75	39	Liver
Set 1/b1	M47	F(K)	0/75	39	Liver
Set 1/b2	M571	800	4/5	234	Liver
Set 1/b3	M795	800	4/5	234	Liver
Set 1/b4	M815	800	6	312	Liver
Set 1/b5	M025	B	5	260	Liver
Set 1/b6	M310	B	5	260	Liver
Set 1/b7	BM276	B	8	416	Liver
Set 1/b8	BM863	B	21	1092	Liver
Set 1/b9	BM555	11b	10	520	Liver
Set 1/b10	M056	Omega	6	312	Liver
Set 1/b11	M098	Omega	5	260	Liver
Set 1/b12	M033	B	6/5	338	Liver
Set 2/A3	F033	B	5	260	Liver
Set 2/A4	F033	B	5	260	Skin
Set 2/A5	F368	B	5	260	Liver
Set 2/A6	F368	B	5	260	Skin
Set 2/A7	F 279	800	5	260	Liver
Set 2/A8	F 279	800	5	260	Skin
Set 2/A9	F619	Omega	6	312	Liver
Set 2/A10	F619	Omega	6	312	Skin
Set 2/A11	F099	CF27	4	208	Liver
Set 2/A12	F099	CF27	4	208	Skin

Set 2/B1	BF336	11b	7	364	Liver
Set 2/B2	BF336	11b	7	364	Skin
Set 2/B3	BF7095	B	21	1092	Liver
Set 2/B4	BF7095	B	21	1092	Skin
Set 2/B5	F198	NN	23	1196	Liver
Set 2/B6	F198	NN	23	1196	Skin
Set 2/B9	M2	11C	1/5	78	Liver
Set 2/B10	M2	11C	1/5	78	Skin
Set 2/1B11	M191	N	4	208	Liver
Set 2/B12	M191	N	4	208	Skin
Set 2/C1	M775	G	9	468	Liver
Set 2/C2	M775	G	9	468	Skin
Set 2/C5	F353	11a	0/83	43	Liver
Set 2/C6	F353	11a	0/83	43	Skin
Set 2/C7	F046	11a	0/83	43	Liver
Set 2/C8	F046	11a	0/83	43	Skin
Set 2/C9	F581	G	11	572	Liver
Set 2/C10	F581	G	11	572	Skin
Set 2/C11	F264	G	11	572	Liver
Set 2/C12	F264	G	11	572	Skin
Set 2/D1	M285	11A	2	104	Liver
Set 2/D2	M285	11A	2	104	Skin
Set 2/D5	M612	11A	2	104	Liver
Set 2/D6	M612	11A	2	104	Skin
Set 2/D7	M353	CF05A	11	572	Liver
Set 2/D8	M353	CF05A	11	572	Skin
Set 2/D9	M124	CF05A	10	520	Liver
Set 2/D10	M124	CF05A	10	520	Skin
Set 2/D11	F10	Zoo	19	988	Liver
Set 2/D12	F10	Zoo	19	988	Skin

Animal number prefixes as follows: BM, breeding male; M, non-breeding male; F, non-breeding female; BF, breeding queen.

Supplementary Table 2. Primer sequences used for targeted assay of methylation.

Primer ID	Chr	Spos	Epos	Forward primer	Reverse primer
NMR 2	JH602048	1786748	1787040	ACACTGACGACATGGTTCTACAt gtgtgaaaaaTaagagtggtggt	TACGGTAGCAGAGACTTGGT CTAtttcctcccttcctcctAaca
NMR 4	JH602120	4015571	4015913	ACACTGACGACATGGTTCTACA gggaggaaggTttTagagatggg	TACGGTAGCAGAGACTTGGT CTAcaccttcctcaccccaAAca
NMR 5	JH602160	1882997	1883289	ACACTGACGACATGGTTCTACA ggtgggtggggtgaaagtag	TACGGTAGCAGAGACTTGGT CTAactccccaaccactaActcct
NMR 6	JH602123	5429096	5429239	ACACTGACGACATGGTTCTACA agagggtTaTatggagtggttTTT	TACGGTAGCAGAGACTTGGT CTAacctttAattcaccaAaAccttttct
NMR 7	JH602136	8746392	8746577	ACACTGACGACATGGTTCTACA gagtTtgggtgggagTtgtT	TACGGTAGCAGAGACTTGGT CTAAccccccttAActcaAttccca
NMR 8	JH602050	17076305	17076585	ACACTGACGACATGGTTCTACA aggTtggaTattttaggaagtgtgT	TACGGTAGCAGAGACTTGGT CTAAtattcctaataaAcccaAActttcca
NMR 9	JH602201	46883	47029	ACACTGACGACATGGTTCTACA TtgtaTTTtgTaaTTTTtgTaggtggg	TACGGTAGCAGAGACTTGGT CTAccctAaAcccaacaccctctc
NMR 12	JH602080	19159913	19160063	ACACTGACGACATGGTTCTACA TaggggTaggTtgtgggagg	TACGGTAGCAGAGACTTGGT CTActcactccaccctcaccaac
NMR 13	JH602069	15422990	15423216	ACACTGACGACATGGTTCTACA gttgTTTTagTtgggTaTtgtaggt	TACGGTAGCAGAGACTTGGT CTAActctAcctctAAccaAAccaAcc
NMR 14	JH602048	11061391	11061687	ACACTGACGACATGGTTCTACA aggggTagTtgggTTaggTT	TACGGTAGCAGAGACTTGGT CTAtctcaccacaAtAcccccaA
NMR 15	JH602044	?	?	ACACTGACGACATGGTTCTACA gggaaTTaggagTtggagggg	TACGGTAGCAGAGACTTGGT CTAacacctttcaaaaccaAaatttcct
NMR 16	JH602051	26386554	26386712	ACACTGACGACATGGTTCTACA gaggggagttggggtgaatgt	TACGGTAGCAGAGACTTGGT CTAAcccaccaaacatcaaccct

Chr: chromosome/contig. position, Spos: start position, Epos: end position, when mapped to the naked mole rat genome (https://www.ncbi.nlm.nih.gov/pubmed/25172923). NMR15 could not be unambiguously mapped to a position on JH602044. The first part of the primer sequences in upper case are the common adapter sequence for fluidigm C1 amplification. Upper case bases in the target sequences that follow are positions which would be Cs in the reference, but as these primers are used against bisulfite converted DNA, they have been changed to Ts (or As in the case of the reverse primers).

Supplementary Table 3. 23 NMR aDMPs identified from the 51 CpGs with chromosome/contig. position (Chr) and position, when mapped to the naked mole rat genome (https://www.ncbi.nlm.nih.gov/pubmed/25172923).

	Chr	Position
1	JH602136	8746439
2	JH602136	8746449
3	JH602136	8746436
4	JH602136	8746451
5	JH602136	8746445
6	JH602048	1786884
7	JH602048	1786857
8	JH602048	1786864
9	JH602136	8746467
10	JH602080	19159979
11	JH602048	1786873
12	JH602048	1786866
13	JH602136	8746485
14	JH602048	1786879
15	JH602201	46935
16	JH602123	5429210
17	JH602136	8746420
18	JH602080	19160007
19	JH602048	1786845
20	JH602123	5429173
21	JH602048	1786852
22	JH602136	8746480
23	JH602048	1786834
